# Factors associated with poor asthma symptom control in adult Angolan regularly seen at an outpatient respiratory clinic

**DOI:** 10.4314/ahs.v23i3.78

**Published:** 2023-09

**Authors:** Margarete L T Arrais, Tiago J P Maricoto, Ofélia M Lulua, Francisca G S Quifica, Jorge M R Gama, Miguel D Brito, Luis Taborda-Barata

**Affiliations:** 1 Department of Pulmonology, Military Hospital Luanda, Angola; 2 Centro de Investigação em Saúde de Angola - CISA, Caxito, Bengo, Angola; 3 Aveiro Healthcare Centre, Aradas Family Health Unit, Aveiro, Portugal; 4 Faculty of Health Sciences, University of Beira Interior, Covilhã, Portugal; 5 UBIAir - Clinical & Experimental Lung Centre, UBIMedical, University of Beira Interior, Covilhã, Portugal; 6 Centre of Mathematics and Applications, Faculty of Sciences, University of Beira Interior, Covilhã, Portugal; 7 Health and Technology Research Centre - H&TRC, Escola Superior de Tecnologia da Saúde de Lisboa, Instituto Politécnico de Lisboa, Portugal; 8 Department of Allergy & Clinical Immunology, Cova da Beira University Hospital Centre, Covilhã, Portugal

**Keywords:** Asthma, Angola, symptom control, inhalers

## Abstract

**Background:**

Asthma is one of the most common chronic respiratory diseases and one of the most frequent causes of hospital care.

**Objectives:**

To describe the clinical characteristics of asthma and factors associated with its control.

**Methods:**

A cross-sectional study was conducted at the Military Hospital in Luanda, from April 2018 to March 2019. Data collection was performed using questionnaires on asthma symptoms and treatment, socio-demographic and environmental questions, and a Global Initiative for Asthma (GINA) questionnaire to assess the level of asthma control. Ordinal logistic regression analyses were performed. We estimated odds ratios, for higher categories of asthma control. P<0.05 was considered significant.

**Results:**

The sample consisted of 305 asthmatics ≥18-years-old, 56% women, with a mean age of 41.3 years. About 28% of patients had controlled asthma, 36% partially controlled and 35% uncontrolled. Poor asthma control was associated with frequent use of short-acting beta-2 agonists [OR 5.70 (95%CI 2.37;13.7)], oral corticosteroids [OR 3.68 (95%CI 2.24;6.04)], and incorrect inhaler technique [OR 4.08 (95%CI 1.25;13.3)].

**Conclusions:**

A significant number of adults living in Luanda have uncontrolled asthma due to the under-use of inhaled corticosteroid therapy. It is necessary to develop strategic management and prevention plans to improve Angolan asthmatics' medical care.

## Introduction

Asthma is one of the most common chronic respiratory diseases and affects all ages. It is a public health problem because it is one of the most frequent causes of hospital care. Asthma attacks are responsible for most asthma morbidity, use of health services, and poor quality of life, and precede most asthma deaths^1^. The burden of asthma worldwide is significant because even in developed countries asthma mortality is still a problem and in many cases is associated with preventable factors^2^. Global asthma mortality is estimated at 0.19/100,000 inhabitants, but there are significant disparities among countries, with low- and middle-income countries with the highest number of asthma deaths^3^.

Data on asthma and its determinants in African adults are scarce. This may be related to difficulties in defining and diagnosing asthma (reported asthma versus medical diagnosis), and may also be due to problems in accessing health care and inhaled treatment. Existing African data show a variable, but globally low, prevalence among different countries: 3.8% in South Africa, 2.0% in Nigeria, 3.3% in Tanzania, 2.7% in Cameroon, and 2.9% in the Democratic Republic of Congo^4^–^6^. In addition, risk factors, exacerbations, and mortality rates are associated with socio-economic level, poor health service conditions, poor asthma control, and access to treatment, and these are factors that need to be addressed^7^,^8^.

Studies performed in children and adolescents in Angola showed a prevalence of up to 15% and some relevant risk factors have been identified^9^,^10^. Nevertheless, a more detailed and comprehensive understanding is needed regarding clinical features and key factors associated with asthma control in adults. Thus, this study aimed to evaluate and describe the clinical characteristics of asthma and factors associated with its control, as well as the use of inhaled therapy, in adults followed in an outpatient pulmonology clinic.

## Methods

### Study design, study area, and sample

A cross-sectional study was conducted at Military Hospital in Luanda from April 2018 to March 2019, with adult patients followed in the outpatient pulmonology clinic. The sample consisted of patients aged 18-years-old and over, with a diagnosis of asthma, according to the Global Initiative for Asthma (GINA) criteria^11^. The sample size was determined according to Peduzzi's rule-of-thumb for multiple logistic regression indicated that a sample size of 270 would be necessary if 7 variables and a prevalence of 0.3 for controlled asthma (the lowest prevalence among the three categories) were considered^12^. Therefore, it was decided to include at least 300 patients in our study. Patients with tuberculosis (TB), pulmonary sequelae, and Chronic Obstructive Pulmonary Disease (COPD) were excluded.

### Data collection and instruments

#### Questionnaires

Data collection was performed using questionnaires validated in Portuguese, related to asthma symptoms, personal and family history, drug use, socio-demographic and environmental questions. Questionnaires also included questions relating to the level of education and socio-economic level, including the occupation, source of family income, type and comfort in housing as well as the characteristics of the place of residence (GRAF-FAR international classification)^13^. GINA questionnaire was used to assess the level of asthma control^11^. To evaluate treatments used, patients answered a questionnaire of nine multiple-choice questions about the type of drug they used in crisis and for maintenance [short-acting beta-2 agonist (SABA), long-acting beta-2 agonist (LABA), inhaled corticosteroid (ICS) or none]; if they received initial training for inhaler technique and whether it was regularly checked. Then patients demonstrated inhaler technique with their devices. Inhaler technique was evaluated according to a checklist, based on the instructions of the National Authority of Medicine and Health Products of Portugal (Infarmed) for pressurized inhalers (pM-DI-pressurized Metered Dose Inhaler) and powder inhalers (Turbohaler®-TH; Diskus®-DK; Aerolizer®-ARL; Breezhaler®-BZL)^14^. Inhaler technique was classified as correct when no error was observed, acceptable when at least one minor error was observed and incorrect when at least one major error was observed. Patients who used different types of inhalers took the same steps for the second inhaler, as performed in other studies^15^.

Data were obtained by interviews performed by physicians specifically trained for this purpose, before or after the outpatient clinical appointment, and complemented with data from clinical files.

#### Definitions

Asthma was defined by the presence of recurrent episodes of dyspnea, chest tightening, wheezing, and/or the use of asthma medication in the last 12 months and medical diagnosis of asthma^11^. Asthma control was based on GINA guidelines^11^, where adequate asthma control should include minimal or absent daytime and night-time symptoms, absence of limitation to physical activity, and minimum need to use rescue medication. With these parameters, GINA classifies control-based asthma as controlled, partially controlled, and uncontrolled^11^.

#### Spirometry

Respiratory function was assessed by spirometry and bronchodilation test performed by two cardiopulmonology technicians, supervised by a pulmonologist. A Spirolab III spirometer (MIR-Medical International Research, Rome, Italy) was used. Reference values used were those standardized by 2005 American Thoracic Society/European Respiratory Society (ATS/ERS) guidelines, which classify obstruction as mild, moderate, moderately severe, severe, and very severe, based on a percentage of the predicted value of Forced Expiratory Volume in the first second (FEV1), as a parameter to assess the severity of airway obstruction in mild, moderate and severe, as well as the presence of response to bronchodilator if there is an increase equal or greater than 200 ml and 12%, after inhalation of a short-acting beta-2 agonist^16^. After recording the personal identification data and anthropometric parameters, patients underwent baseline spirometry. The choice of manoeuvres was made according to the ATS/ERS criteria based on the highest values of Forced Expiratory Volume (FEV1) and Forced Vital Capacity (FVC) obtained at least three acceptable manoeuvres in which at least two of them were reproducible. Then fifteen minutes after inhalation of 400 µg salbutamol per expander chamber, patients repeated ventilatory manoeuvres to assess response to bronchodilator^16^.

#### Ethical considerations

This study was approved by the Ethics Committee of the Military Hospital in Luanda (date of approval: April 3, 2018). All patients who agreed to participate in the study gave written informed consent. After the interview, all patients received basic information about asthma, the use of inhalers and the correct technique for their use was shown.

#### Statistical analysis

Asthma control was considered the main outcome and classified in accordance to the GINA guidelines in controlled, partially controlled and uncontrolled^11^. Data were analysed using the Statistical Package for the Social Sciences (SPSS), version 25.0 software. Descriptive analysis was used for sample characterisation. Ordinal logistic regression analyses with logit cumulative link function were performed to determine the independent factors that predicted asthma control. We estimated the odds ratio, non-adjusted (OR) or adjusted (aOR), for higher categories of asthma control. To build the models we first performed bivariate associations to identify significant variables to be included at a 0.1 alpha level^17^. Models were set using step-up and step-down approaches, and different models were rechecked with homoscedasticity tests. A two-tailed P value < 0.05 was considered significant.

## Results

### General sample characteristics

Three hundred forty-nine patients with asthma diagnoses were sequentially recruited to participate in the study. Of these, 44 were excluded for various reasons (5 did not agree to participate, 16 for traumatic or TB sequelae, 23 for unconfirmed diagnosis, and/or did not complete the study).

The studied sample consisted of 305 asthmatics aged 18 years or older, 56% were women, 65% were active workers, 44% had studied between ten and twelve years, and 45% belonged to Graffar IV and V social class, 88.2% had never smoked (Table 1).

**Table 1:** General characteristics of the sample (N=305)

Age (years)	Mean ± SD Median (min; max)	41.3 ± 15.5 41 (18; 86)
Gender n	Women	171 (56.1)
(%)	Men	134 (43.9)
BMI	Mean ± SD	26.7 ± 9.0
(Kg/m^2^)	Median (min; max)	25 (15.2; 137.1)
Profession n	Not working	74 (24.3)
(%)	Working / active	199 (65.2)
	Pensioners	32 (10.5)
Schooling n	No schooling	17 (5.6)
(%)	4 years	8 (2.6)
	5-9 years	49 (16.1)
	10-12 years	133 (43.6)
	> 12 years	98 (32.1)
Graffar n	Class I	2 (0.7)
(%)	Class II	42 (13.8)
	Class III	124 (40.7)
	Class IV	115 (37.7)
	Class V	22 (7.2)
Smoking n	Never smoked	269 (88.2)
(%)	Ex-smoker	36 (11.8)

BMI: Body Mass Index

### Asthma control

Of the 305 patients included in the study, 28.2% had controlled asthma, 36.4% partially controlled, and 35.4% uncontrolled, with no significant differences in gender, age, and BMI. Poor asthma control was significantly associated with some precipitating factors such as household dust, viral respiratory infections, exercise, temperature variations, and strong emotions, but the adjusted OR confirmed that only exercise was significantly related to poor asthma control (Table 2). Ninety-two patients (30.2%) had five or more exacerbations, 65 (21.3%) had one or two hospitalizations, and 12 (3. %) had five or more hospitalizations in the last year.

**Table 2:** Asthma control and precipitating factors

	Controlled	Partially controlled	Uncontrolled	OR for worse control	P^a^	aOR for worse control	P^b^
	(n = 86)	(n = 111)	(n = 108)	(95% CI)		(95% CI)	
Age (years)							
Mean ± SD	44.1±15.4	36.8±14.9	43.4±15.5	1.00(0.98;1.01)	0.951	-	-
Median (min; max)	47 (18; 86)	35 (18; 85)	41.5 (18; 86)				
Gender, n (%)							
Men	40 (46.5)	46 (41.4)	48 (44.4)	1		-	-
Women	46 (53.5)	65 (58.6)	60 (55.6)	1.04(0.69;1.58)	0.825		
BMI (kg/m^2^)							
Mean ± SD	27.0±6.7	27.1±12.1	26.2±6.6	0.99(0.97;1.01)	0.603	0.97(0.94;1.00)	0.077
Median	25.7	24.9	25.0				
(min;max)	(15.4; 47.4)	(15.2; 137.1)	(15.5; 64.5)				
Smoking, n (%)							
Never smoked	76 (88.4)	104 (93.7)	89 (82.4)	1		1	
Ex-smoker	10 (11.6)	7 (6.3)	19 (17.6)	1.73(0.99;3.33)	0.099	2.65(0.96;7.31)	0.060
Tobacco exposure, n (%)							
No	74 (86.0)	97 (87.4)	93 (86.1)	1		-	-
Yes	12 (14.0)	14 (12.6)	15 (13.9)	1.00(0.54;1.84)	0.984		
Animals at home, n (%)							
No	38 (44.2)	57 (51.4)	44 (40.7)	1		-	-
Yes	48 (55.8)	54 (48.6)	64 (59.3)	0.87(0.57;1.32)	0.530		
Aerosols, n (%)							
Without exposure	7 (8.1)	3 (2.7)	4 (3.7)	1			
Non-precipitating exposure	8 (9.3)	8 (7.2)	6 (5.6)	1.41(0.40;4.91)	0.585	-	-
Precipitating exposure	71 (82.6)	100 (90.1)	98 (90.7)	2.20(0.80;6.01)	0.123		
Intense smells, n (%)							
Without exposure	8 (9.3)	3 (2.7)	7 (6.5)	1			
Non-precipitating exposure	8 (9.3)	8 (7.2)	6 (5.6)	0.94(0.29;2.96)	0.918	-	-
Precipitating exposure	70 (81.4)	100 (90.1)	95 (88.0)	1.44(0.59;3.47)	0.413		
Animals with fur, n (%)							
Without exposure	64 (74.4)	77 (69.4)	86 (79.6)	1			
Non-precipitating exposure	18 (20.9)	20 (18.0)	15 (13.9)	0.69(0.40;1.21)	0.202	-	-
Precipitating exposure	4 (4.7)	14 (12.6)	7 (6.5)	1.01(0.47;2.17)	0.970		
Feathered animals, n (%)							
Without exposure	82 (95.3)	108 (97.3)	105 (97.2)	1			
Non-precipitating exposure	4 (4.7)	2 (1.8)	3 (2.8)	0.62(0.18;2.13)	0.455	-	-
Precipitating exposure	0 (0.0)	1 (0.9)	0 (0.0)	0.83(0.02;30.8)	0.922		
Household dust, n (%)							
Without exposure	47 (54.7)	52 (46.8)	45 (41.7)	1			
Non-precipitating exposure	26 (30.2)	23 (20.7)	19 (17.6)	0.80(0.47;1.37)	0.429	-	-
Precipitating exposure	13 (15.1)	36 (32.4)	44 (40.7)	2.23(1.36;3.65)	0.001		
Viral resp. infections, n (%)							
Rarely	42 (48.8)	34 (30.6)	19 (17.6)	1			
Not precipitating	3 (3.5)	4 (3.6)	5 (4.6)	2.67(0.87;8.19)	0.084	-	-
Precipitating	41 (47.7)	73 (65.8)	84 (77.8)	2.98(1.87;4.76)	<0.001		
Temperat. variations, n (%)							
Without exposure	18 (20.9)	14 (12.6)	9 (8.3)	1			
Non-precipitating exposure	12 (14.0)	7 (6.3)	5 (4.6)	0.82(0.31;2.11)	0.681	-	-
Precipitating exposure	56 (65.1)	90 (81.1)	94 (87.0)	2.44(1.31;4.55)	0.005		
Exercise, n (%)							
Does not exercise	39 (45.3)	39 (35.1)	25 (23.1)	1		1	
Not precipitating	34 (39.5)	33 (29.7)	11 (10.2)	0.69(0.39;1.19)	0.188	0.45(0.18;1.13)	0.091
Precipitating	13 (15.1)	39 (35.1)	72 (66.7)	4.81(2.86;8.08)	<0.001	2.31(1.07;4.99)	0.032
Strong emotions, n (%)							
Rarely	44 (51.2)	63 (56.8)	37 (34.3)	1		1	
Not precipitating	26 (30.2)	15 (13.5)	13 (12.0)	0.60(0.33;1.08)	0.089	1.29(0.49;3.39)	0.602
Precipitating	16 (18.6)	33 (29.7)	58 (53.7)	2.98(1.84;4.83)	<0.001	1.89(0.91;3.92)	0.084
Drugs (aspirin, non-steroidal antiinflammatory, beta-blockers), n (%)							
Rarely use							
Not precipitating	85 (98.8)	106 (95.5)	105 (97.2)	1			
Precipitating	1 (1.2)	1 (0.9)	1 (0.9)	0.85(0.10;6.92)	0.883	-	-
	0 (0.0)	4 (3.6)	2 (1.9)	1.60(0.36;7.30)	0.541		

a Wald test

b Wald test

Likelihood ratio test, P < 0.001; Parallel lines test, P = 0.108; Nagelkerke R^2^=81.2%

BMI: Body Mass Index OR: Odds ratio; aOR: Adjusted Odds ratio; CI: Confidence Interval; P: P-value; Temperate: temperature

### Spirometry

All patients performed spirometry before and after a short-acting beta-2 agonist (salbutamol). It was normal in 66 (21.6%) patients, and showed mild obstruction in 146 (47.9%), moderate to severe in 67 (22.0%), and severe to very severe in 26 (8.5%) patients. Of 239 patients whose spirometry showed obstruction, the bronchodilator test was positive in 122 (51.0%). Severe/very severe obstruction was significantly associated with poor asthma control [aOR 14.660 (95%CI2.604;82.525) p=0.002]

### Drugs used in asthma treatment

One hundred and ninety-four (63.6%) patients used some type of inhaler, either a pressurised metered-dose inhaler (pMDI) and/or a dry powder inhaler (DPI). We observed that 159 (52.1%) patients used only SABA either inhaled and/or oral, and some patients used it as relief treatment, but others also used maintenance. One hundred and twenty-one (39.7%) used inhaled corticosteroids (ICS). Of these, 71 (58.7%) patients used ICS associated with LABA and 50 (41.3%) used ICS and SABA relief, however irregularly. Inhaler technique in pMDI and DPI was incorrect in 65.7% and 54.4%, respectively, although 94.3% of patients, answered affirmatively that they had training on the inhaler technique but only 21.4% of them confirmed regular verification of the technique. Two hundred and twenty-one (72.5%) patients had used or often used oral corticosteroids, especially in exacerbations (Table 3). Sixty-three (20.7%) patients also used leukotriene receptor antagonists and 39 (12.8%) used theophylline, and these patients used SABA relief.

**Table 3:** Asthma control and drugs used

	Controlled (n = 86)	Partially controlled (n = 111)	Uncontrolled (n = 108)	OR for worse control (95% CI)	P^a^
Short-acting beta-2					
agonist					
No	57 (66.3)	55 (49.5)	34 (31.5)	1	
Yes inhaled	20 (23.3)	39 (35.1)	47 (43.5)	2.67(1.66;4.29)	<0.001
Yes oral	7 (8.1)	10 (9.0)	12 (11.1)	2.21(1.05;4.65)	0.036
Yes, inhaled and oral	2 (2.3)	7 (6.3)	15 (13.9)	5.70(2.37;13.7)	<0.001
Long-acting beta-2					
agonist					
No	62 (72.1)	97 (87.4)	75 (69.4)	1	
Yes	24 (27.9)	14 (12.6)	33 (30.6)	1.23(0.75;2.01)	0.401
Inhaled corticosteroid					
No	32 (37.2)	85 (76.6)	67 (62.0)	1	
Yes	54 (62.8)	26 (23.4)	41 (38.0)	0.49(0.32;0.76)	0.001
Observed pMDI use					
(n=134)					
Correct technique	2 (7.7)	7 (15.2)	4 (6.5)	1	
Acceptable technique	9 (34.6)	11 (23.9)	13 (21.0)	0.97(0.29;3.18)	0.769
Incorrect technique	15 (57.7)	28 (60.9)	45 (72.6)	0.94(0.29;3.06)	0.924
Observed DPI use					
(n=114)					
Correct technique	9 (17.3)	3 (12.5)	2 (5.3)	1	
Acceptable technique	20 (38.5)	13 (54.2)	5 (13.2)	1.39(0.40;4.83)	0.601
Incorrect technique	23 (44.2)	8 (33.3)	31 (81.6)	4.08(1.25;13.3)	0.019
Inhaler technique					
training					
(n=194)					
Yes	62 (98.4)	53 (93.0)	68 (91.9)	0.41(0.12;1.35)	0.144
No	1 (1.6)	4 (7.0)	6 (8.1)	1	
Regular verification of					
inhaler technique					
(n=192)					
Yes	22 (34.9)	7 (12.5)	12 (16.4)	0.41(0.21;0.79)	0.008
No	41 (65.1)	49 (87.5)	61 (83.6)	1	
Oral corticosteroid					
No	13 (15.1)	21 (18.9)	50 (46.3)	1	
Yes	73 (84.9)	90 (81.1)	58 (53.7)	3.68(2.24;6.04)	<0.001

a Teste de Wald

pMDI: pressurized metered-dose inhaler; DPI: Dry powder inhaler; OR: Odds ratio; CI: Confidence Interval; P: P-value

Poor asthma control was significantly associated with frequent use of short-action beta-2 agonist (about 2 to 6-fold), use of oral corticosteroids (about 4-fold), and as well as incorrect inhaler technique of inhaled DPI devices (about 4-fold), while inhaled corticosteroid use was significantly associated with better control (Table 3).

Correct and acceptable inhaler technique, was not associated with training or with regular verification of pMDI or DPI technique (Table 4).

**Table 4:** Inhaler technique and inhaler training of pMDI and DPI

Observed pMDI inhaler use	Correct (n = 13)	Acceptable (n = 33)	Incorrect (n = 88)	Adjusted OR (95% CI)	P^(1)a^
Inhaler technique training (n=134)					
Yes	13(100.0)	30 (90.9)	81 (92.0)	1.16(0.47;2.87)	0.737
No	0 (0.0)	3 (9.1)	7 (8.0)	1	
Verification of inhaler technique (n=133)					
Yes	5 (38.5)	8 (24.2)	9 (10.3)	0.88(0.42;1.84)	0.742
No	8 (61.5)	25 (75.8)	78 (89.7)	1	

Observed DPI inhaler use	Correct (n = 14)	Acceptable (n = 38)	Incorrect (n = 62)	Adjusted OR (95% CI)	P^(2)a^

Inhaler technique training (n=114)					
Yes	14(100.0)	38 (100.0)	59 (95.2)	-	-
No	0 (0.0)	0 (0.0)	3 (4.8)		
Verification of inhaler technique (n=113)					
Yes	9 (64.3)	8 (21.1)	15 (24.6)	0.51(0.25;1.03)	0.064
No	5 (35.7)	30 (78.9)	46 (75.4)	1	

(1)a Wald ‘test; Likelihood ratio test, P=0.001; Test of parallel lines, P=0.359; Nagelkerke R^2^=15.8%

(2)a Wald ‘test; Likelihood ratio test, P=0.227; Test of parallel lines, P=0.086; Nagelkerke R^2^=4.4%

pMDI: pressurized metered-dose inhaler; DPI: Dry powder inhaler; OR: Odds ratio; CI: Confidence Interval; P: P-value

## Discussion

This study on asthma in Angolan adults showed that most patients have uncontrolled asthma, a large number of them had several exacerbations and used oral corticosteroids and SABA in the last year. A small number of patients used inhaled corticosteroids and most patients had incorrect inhaler techniques. Poor asthma control was associated with frequent use of SABA, use of oral corticosteroids, and incorrect inhaler technique, while ICS use was associated with better asthma control.

Patients were mostly women with an average age ranging from 30 to 40 years, and low socioeconomic level. Asthma in young individuals is generally related to clinical manifestations of childhood-onset, as shown in studies with a high prevalence of asthma in children in Luanda^10^. Low socio-economic level, present in near half of patients in our study, suggests that poor asthma control may be linked to limitations in access to health care, inhaler drugs, or even to some housing or environmental conditions, such as exposure to indoor and/or outdoor pollution, as described in other studies^5^,^6^,^8^,^18^, but also to geographical factors and genetic susceptibility^19^.

Some studies conducted in low-to-middle-income countries (LMIC) have shown a high number of acute asthma patients, and that is influenced by problems in access to health care^8^,^20^,^21^, low adherence to maintenance ICS treatment8,18 and also climate variations^18^. Thirty percent of patients who participated in our study had five or more exacerbations and 21% had one or two hospitalisations for asthma last year. We also found a considerable number (47%) of patients who had used oral corticosteroids in the last year, which may reflect the severity of exacerbations or even the lack of access to maintenance inhaler therapy.

Only 28% of the patients had controlled asthma, while the remaining had partially controlled or uncontrolled asthma. Although numerous factors are related to asthma exacerbations and their control^22^, we found no relationship between poor control and several precipitating factors, such as active and passive smoking, household and environmental factors, viral respiratory infections, strong emotions, and use of specific drugs. The exception is exercise, which proved to be a significant precipitating factor.

Poor asthma control was significantly associated with frequent use of short-action beta-2 agonist, use of oral corticosteroids, and as well as incorrect inhaler technique of DPI devices, while the use of continuous ICS treatment was associated with better control, as reported in several studies^23^,^24^.

Incorrect inhaler technique has been described as one of the relevant causes of poor asthma control25,26, and we found a similar trend, although most participants had already received initial training in inhaler technique. Lack of treatment is one of the causes of inadequate asthma control. In this study, we observed that more than half of patients used short-acting beta-2 agonist (inhaled and per os), either as a rescue or an as a regular treatment. This widespread use, even though not prescribed by a doctor most times, is commonly observed because it provides quick relief of symptoms, but also because prescription of controller treatment is not that frequent, and is frequently expensive, as concluded by some studies in LMIC27. Less than half of the patients use ICS (associated or not with a LABA or SABA), and this may explain why poor control is significantly related to frequent use of SABA and oral corticosteroids, as shown in other studies^8^,^18^,^20^,^21^.

This study has several limitations. First, the sample should be wider and multicentre, including patients from various hospitals in the country, thereby producing more comprehensive and representative results of Angolan asthmatics. Secondly, our study only focused on patients seen at a specialty hospital outpatient unit and therefore may not fully represent the situation of asthma in the general community, where underdiagnosis, under-treatment, and lack of control may be even more prevalent, as our group observed in children and adolescents of the general community^9^,^10^. Thirdly, although our results were obtained using validated questionnaires and techniques, some may be biased by patients' self-report. However, we tried to minimise these aspects by confirming all data retrospectively through the records of each patient's clinical files. In addition, it was not possible to determine actual patient adherence to prescribed treatments in a rigorous and validated form, as we only used patient information. Despite these limitations, this study on asthma in adults conducted in Angola, whose results have public health impact by showing that priority actions are needed to improve the diagnosis and treatment of this disease. Our results were obtained with validated questionnaires and techniques, so they can serve as a basis for further studies with more comprehensive approaches in other provinces of the country, as well as for implementation of strategies for treatment and control of asthma in the country.

## Conclusions

A significant number of asthmatic adults living in Luanda had not fully controlled asthma, due to the under-use of inhaled corticosteroid therapy. It is necessary to develop strategic management and prevention plans to improve Angolan asthmatics' medical care. National guidelines for asthma management are needed.
